# Supplementary material for: Enhanced Resistance of atbzip62 against Pseudomonas syringae pv. tomato Suggests Negative Regulation of Plant Basal Defense and Systemic Acquired Resistance by AtbZIP62 Transcription Factor
Source: Int J Mol Sci. 2021 Oct 26;22(21):11541. doi: 10.3390/ijms222111541 (PMC8584143; doi:10.3390/ijms222111541)
Supplement: Supplementary file 1 [file ijms-22-11541-s001.zip › ijms-1387930-supplementary.pdf]

Table S1. List of genes and their corresponding primers used in the study.

S.No	Gene Name	Accession No.	Forward sequence	Reverse sequence
1	*AtActin*	AT3G18780	GCTGGACGTGACCTTACTGA	CCATCTCCTGCTCGTAGTCA
2	*AtPR1*	AT2G14610	GTGCAATGGAGTTTGTGGTC	TCACATAATTCCCACGAGGA
3	*AtPR2*	AT3G57260	CAGATTCCGGTACATCAACG	AGTGGTGGTGTCAGTGGCTA
4	*AtG3PDH*	AT2G41540	AAATATGTCGAGGCAAGGCT	CCACACAGCTTCTTGCAGAT
5	*AtAZI*	AT4G12470	GCAAGCCAAGTCCTAAACCA	GTCGACGTCAACCAAACCTT
